# An Unusual Movement Disorder—Case of Diabetic Striatopathy

**DOI:** 10.1210/jcemcr/luae201

**Published:** 2024-10-25

**Authors:** Shazia Azmat, Owais Lodhi, Harish Ashok, Hussein Harb, Mahwash Siddiqui, Janice Gilden

**Affiliations:** Chicago Medical School, Rosalind Franklin University of Medicine and Science, North Chicago, IL, USA; Department of Internal Medicine, Ascension Illinois-Saint Joseph Hospital, Chicago, IL 60657, USA; Department of Endocrinology, Mount Sinai Hospital, North Chicago, IL 60608, USA; Department of Endocrinology, Mount Sinai Hospital, North Chicago, IL 60608, USA; Department of Endocrinology, Mount Sinai Hospital, North Chicago, IL 60608, USA; Chicago Medical School, Rosalind Franklin University of Medicine and Science, North Chicago, IL, USA

**Keywords:** nonketotic hyperglycemia chorea-ballismus, metabolic syndrome, neuroendocrine

## Abstract

Nonketotic hyperglycemia chorea-ballismus (NKH-CB), a rare metabolic syndrome, arises as a secondary condition to hyperglycemia. It is marked by acute or subacute hemichorea-hemiballismus, hyperglycemic state, and unique reversible striatal abnormalities on neuroimaging. This case presents a 70-year-old Hispanic man with a significant medical history of cerebral vascular accidents, hypertension, bipolar disease, and uncontrolled type 2 diabetes mellitus.

Notably, the patient was experiencing large-amplitude involuntary movements on his left side for the past 3 weeks. With resolution of hyperglycemia, the amplitude and frequency of the involuntary arm movements were absent. This case highlights the need for careful monitoring and tight control of blood glucose levels in patients with a history of diabetes, to prevent serious neurological complications such as NKH-CB syndrome. Prompt diagnosis through neurological evaluation, blood glucose level assessment, and neuroimaging techniques are critical in managing the symptoms effectively.

## Introduction

Nonketotic hyperglycemia chorea-ballismus (NKH-CB), a rare metabolic syndrome, arises as a secondary condition to hyperglycemia [1], mainly seen in elderly Asian women with type 2 diabetes mellitus [2]. Marked by acute or subacute hemichorea-hemiballismus, a hyperglycemic state, and unique reversible striatal abnormalities on neuroimaging [3], NKH-CB is often misdiagnosed due to its uncommonness and its radiographic features, which are easily mistaken for intracerebral hemorrhage [4]. Although the pathophysiology of this syndrome remains largely unclear, evidence suggests that hyperglycemia, ischemia, and microhemorrhages may play a role [5]. The present report introduces an unusual case of simultaneous NKH-CB, history of stroke, and chronic antipsychotic medication use, offering valuable insights into the diagnostic challenges associated with this condition and potential links to other neurological complications of diabetes mellitus, such as stroke, peripheral neuropathy, nonepileptic seizures, and tardive dyskinesia. Additionally, it expands the current understanding of nonketotic hyperglycemia hemichorea-hemiballismus syndrome, a disease characterized by hyperkinetic movements commonly associated with neurodegenerative diseases, including basal ganglia degeneration.

## Case Presentation

The patient is a 70-year-old Hispanic male with a past medical history of 2 cerebrovascular accidents, non-insulin-requiring type 2 diabetes mellitus, hypertension, and bipolar disease. The patient presented to the emergency room with complaints of nonradiating, midsternal chest pain. Vitals were significant for elevated blood pressure of 151/64 mmHg. Laboratory values were significant as indicated in Table 1 below.

**Table 1.** Laboratory values

Labs on admission	Results	Reference range
Blood glucose	447 mg/dL 24.81 mmol/L	< 140 mg/dL; <7.77 mmol/L
A1C%	16.9%	<5.7%
CO_2_	23.6 mEq/L	21.0-31.0 mEq/L
Anion gap	10.0 mmol/L	4-11 mmol/L
Albumin	4.2 g/dL	3.5-5.7 g/dL
Beta hydroxybutyrate	0.2 mmol/L	0.1-0.3 mmol/L
Serum osmolality	322 mosm/kg	280-300 mosm/kg

The physical examination revealed large-amplitude involuntary movements in the left upper and lower extremities and facial region. He also veered to the left when ambulating. There was no facial droop or dysarthria. The patient reported that these abnormal movements were occurring for the last 3 weeks, although sensation was preserved bilaterally.

## Diagnostic Assessment

**Figure 1.**
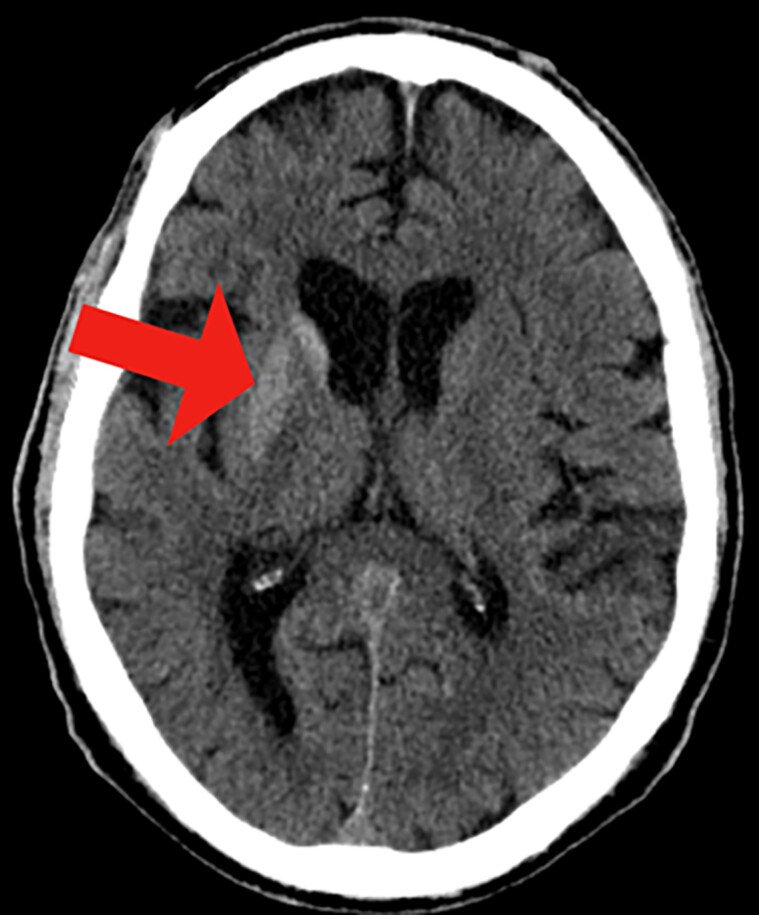
CT displaying unilateral hyperdensity of the right caudate and putamen (red arrow).

Acute coronary syndrome was ruled out with negative serial troponins and normal electrocardiogram. Urgent assessment of stroke was carried out by a noncontrast computed tomography (CT) which showed unilateral hyperdensity of the right caudate and putamen, with no evidence of acute intracranial hemorrhage or large arterial territory infarction (Fig. 1).

A CT angiogram did not show signs of stenosis or occlusion.

**Figure 2.**
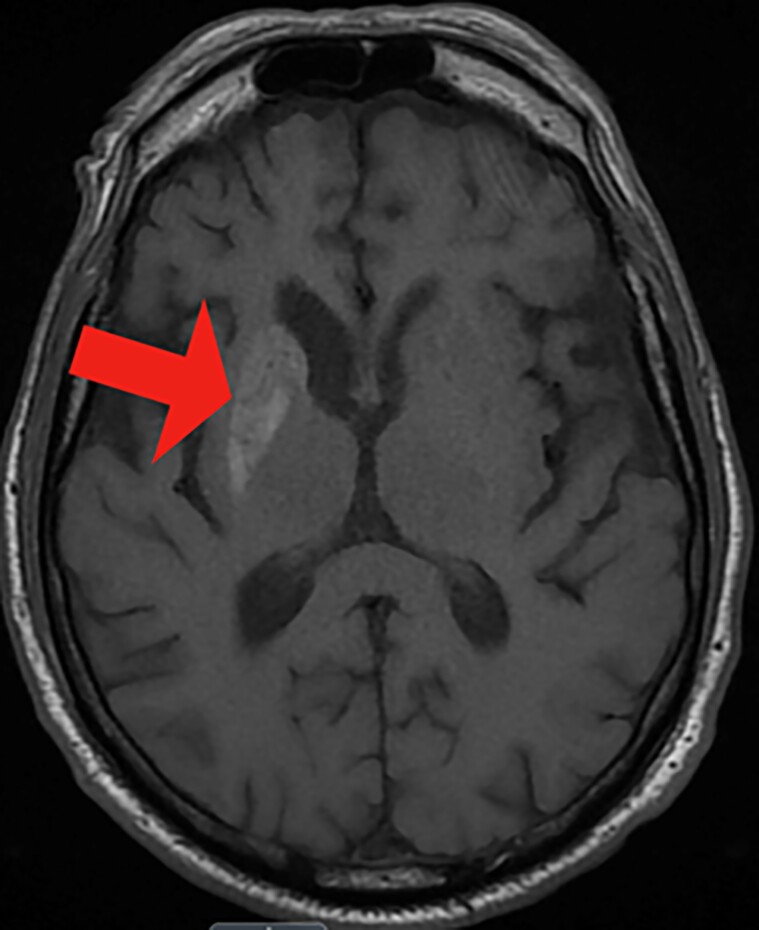
MRI brain: T1 shortening involving the right caudate and putamen corresponding to the hypodensity seen on CT scan (red arrow).

Magnetic resonance imaging (MRI) of the brain showed unilateral T1 hyperintensity involving the right caudate and putamen corresponding to the hypodensity seen on CT (Fig. 2).

Based on the patient's clinical signs and symptoms coupled with neuroimaging, the preliminary diagnosis was nonketotic hyperglycemia hemichorea-hemiballismus syndrome.

## Treatment

The patient was given fluids and 8 units of insulin lispro in the emergency room and admitted for further evaluation. During hospitalization, the patient received insulin basal/bolus regimen with target blood glucose between 140 and 160 mg/dL (7.77-8.88 mmol/L) and risperidone.

## Outcome and Follow-Up

On the second day of admission, with resolution of hyperglycemia, the amplitude and frequency of the involuntary arm movements were absent, confirming her diagnosis. The patient was discharged on basal insulin, metformin 1000 mg (milligrams) daily, as well as risperidone 2 mg daily.

Our patient was lost to follow-up, so no follow-up imaging was conducted at our institution.

## Discussion

This case presents NKH-CB syndrome in patient with poorly controlled type 2 diabetes mellitus, complicated by prior history of recurrent cerebrovascular accidents and bipolar disease, and it highlights the diagnostic challenges with overlap in clinical presentations.

NKH-CB syndrome is a rare complication of poorly controlled diabetes, typically characterized by unilateral limb choreographic dyskinesia, striatal abnormalities on neuroimaging, and nonketotic hyperglycemia [5]. It is often misdiagnosed, as it is uncommon and radiographic features are easily mistaken for intracerebral hemorrhage. Prevalence in the literature is 1 in 100 000, with ethnic variation, and about 77% of cases were reported in elderly Asian women with a mean age of 71 years [6].

The exact pathophysiology of the syndrome remains unclear, but several mechanisms have been proposed. One involves a shift from aerobic to anaerobic metabolism during the pathogenesis of nonketotic hyperglycemia, leading to decreased levels of gamma-aminobutyric acid due to consumption, causing uncoordinated movements [7]. This is in contrast to diabetic ketoacidosis where GABA substrates are synthesized, leading to regional metabolic imbalance and basal ganglia dysfunction.

Several presentations of this condition have been reported with cerebrovascular damage, methamphetamine, antipsychotics, and acute ischemic stroke [8]. Typical exam findings are chorea or ballistic movements. Other neurological manifestations include myoclonus, dystonia, facial spasm, restless leg syndrome, tremors, and Parkinsonism [9].

Typical neuroimaging findings of NKH-CB include striatal hyperdensity on CT scans and striatal hyperintensity on T1-weighted MRI on the contralateral side. In studies, positron emission tomography and single-photon emission computed tomography studies showed hypofunction of striatum, suggesting possible local metabolic dysfunction. However, clinically, this modality has not been much utilized [10].

Our patient was a 70-year-old Hispanic man whose blood glucose level was remarkably high (447 mg/dL) (24.81 mmol/L) (normal reference range: < 140 mg/dL, < 7.77 mmol/L) and A1C was 16.9% (normal reference range: <6.5%) confirming poor glycemic control, which is consistent with disease presentation [11]. MRI brain confirmed hyperdensity in the right caudate and putamen on T1/T2 or diffusion-weighted images, which aligns with the hypodensity seen on CT, supporting the diagnosis of NKH-CB syndrome.

Though our patient has a typical presentation of NKH-CB syndrome, his gender and ethnicity are atypical for NKH-CB syndrome and neurological complications including recurrent strokes, seizures, and tardive dyskinesia were preceding the list of differentials due to his comorbidities.

Insulin therapy and risperidone, along with close monitoring of his blood glucose levels, were implemented immediately. This course of treatment is in line with the current recommendation that insulin therapy reduces the time and severity of involuntary movements [9]. Treatment of NKH-CB syndrome involves tight glycemic control with additional therapies for movement disorders, such as antipsychotics, GABA receptor agonists, selective serotonin uptake inhibitors, and dopamine-depleting agents [12]. In a meta-analysis, successful treatment rate of chorea with only glycemic control was 25.7% compared to 76.2% with addition of antichorea medication [13]. Haloperidol is the most commonly used antichorea medication followed by tetrabenazine, risperidone, and clonazepam.

Seizures are a frequent acute complication of diabetes mellitus and have high rates of morbidity and mortality [14]. Our patient did not have seizures, which are common in NKH patients, as observed in other case discussions [15]. However, it is noteworthy to state that NKH-induced seizures are known to occur in patients older than 50 years [16]. Given presentation of hemichorea-hemiballismus, physicians should remain vigilant for the potential onset of seizures, since diabetes mellitus poses a higher risk [9].

Overall, this case highlights the need for careful monitoring and tight control of blood glucose levels in patients with a history of diabetes, to prevent serious neurological complications such as NKH-CB syndrome. Prompt diagnosis through neurological evaluation, blood glucose level assessment, and neuroimaging techniques are critical in managing the symptoms effectively. Future research is warranted to better understand the pathophysiological mechanisms of the syndrome, particularly in relation to ethnicity and gender, and to develop more targeted treatment strategies.

In summary, NKH-CB syndrome is a unique neurological complication of hyperglycemia that demands a high index of suspicion for prompt diagnosis and management. Understanding the pathophysiology, identifying its characteristic presentation, and familiarizing oneself with the distinctive neuroimaging findings are critical to ensure timely intervention, potentially preventing long-term neurological sequelae. More studies are needed to further elucidate the pathogenesis of this condition, identify predisposing factors, and develop targeted therapies to optimize patient outcomes.

## Learning Points

- Simultaneous NKH-CB, history of stroke, and chronic antipsychotic medications use offers valuable insight into diagnostic challenges associated with this condition and potential links to other neurological conditions.
- Familiarizing oneself with distinctive neuroimaging findings is critical to ensure timely intervention.
- Careful and stringent blood glucose monitoring in patients with diabetes mellitus is key to prevent serious neurological complications.

## Contributors

All authors made individual contributions to authorship. H.H. and H.A. were involved in the diagnosis and management of the patient. O.L. and S.A. were involved in literature review. S.A., M.S., and J.G. were involved in writing and manuscript submission. All authors reviewed and approved the final draft.

## Data Availability

Original data generated and analyzed for this case report are included in this published article.

## Abbreviations

CT: computed tomography
MRI: magnetic resonance imaging
NKH-CB: nonketotic hyperglycemia chorea-ballismus
